# Inbreeding-Driven Innate Behavioral Changes in *Drosophila melanogaster*

**DOI:** 10.3390/biology12070926

**Published:** 2023-06-28

**Authors:** Anusha Amanullah, Shabana Arzoo, Ayesha Aslam, Iffat Waqar Qureshi, Mushtaq Hussain

**Affiliations:** Bioinformatics and Molecular Medicine Research Group, Dow Fly Research Lab and Stock Center, Dow College of Biotechnology, Dow University of Health Sciences, Karachi 75330, Pakistan

**Keywords:** inbreeding, *Drosophila melanogaster*, behavior, inbreeding coefficient, genomic homozygosity

## Abstract

**Simple Summary:**

Inbreeding includes mating between two closely related individuals. It is known to affect the biological fitness of plants and animals, including humans. Experiments conducted on *Drosophila melanogaster*, a common fruit fly, demonstrate adverse effects of inbreeding on reproductive fitness and stress tolerance. However, the impact of inbreeding on the innate behavior of *D. melanogaster* is relatively unexplored. In this study, we bred *D. melanogaster* in a manner to attain different degrees of inbreeding in progeny flies. These flies were then assessed and compared for different behavioral traits. Our findings showed abnormalities in locomotor and phototactic behaviors due to inbreeding. Likewise, changes in aggression and courtship behavior with increasing levels of inbreeding were also observed. Interestingly, among positively phototactic flies, better learning ability was observed in inbred flies compared to outbred flies. Taken together, our study demonstrates that inbreeding influences the innate behavior of *D. melanogaster*. Given the reputation of *D. melanogaster* as one of the most effective animal models, the findings of this investigation could be exploited in animal and livestock breeding, conservation biology and genetic counseling.

**Abstract:**

*Drosophila melanogaster* has long been used to demonstrate the effect of inbreeding, particularly in relation to reproductive fitness and stress tolerance. In comparison, less attention has been given to exploring the influence of inbreeding on the innate behavior of *D. melanogaster*. In this study, multiple replicates of six different types of crosses were set in pair conformation of the laboratory-maintained wild-type *D. melanogaster*. This resulted in progeny with six different levels of inbreeding coefficients. Larvae and adult flies of varied inbreeding coefficients were subjected to different behavioral assays. In addition to the expected inbreeding depression in the-egg to-adult viability, noticeable aberrations were observed in the crawling and phototaxis behaviors of larvae. Negative geotactic behavior as well as positive phototactic behavior of the flies were also found to be adversely affected with increasing levels of inbreeding. Interestingly, positively phototactic inbred flies demonstrated improved learning compared to outbred flies, potentially the consequence of purging. Flies with higher levels of inbreeding exhibited a delay in the manifestation of aggression and courtship. In summary, our findings demonstrate that inbreeding influences the innate behaviors in *D. melanogaster*, which in turn may affect the overall biological fitness of the flies.

## 1. Introduction

Inbreeding, a form of mating between genetically related individuals, is a double-edged sword that is known to affect the gene pool of a population. Inbreeding provides benefits through the possible fixation and transfer of advantageous traits and is thus exploited in animal and livestock breeding [1,2]. In contrast, inbreeding could also fix and transfer detrimental and/or disadvantageous alleles in the population. This, in turn, reduces the genetic variations and decreases the fitness of the population in terms of reproduction, survival and general well-being, collectively referred to as inbreeding depression [3]. Adverse consequences of inbreeding in plants, animals and humans have consistently been reported [4,5,6,7,8,9].

Behavior is often one of the key players in the evolutionary success of an organism. A large body of evidence suggests that many of the innate behaviors of organisms have genetic foundations [10,11,12]. Since inbreeding manifests its impact in the accumulation of advantageous and/or disadvantageous alleles, it is therefore conceivable that inbreeding could influence the innate behavior of an organism. Like many genetic studies, the effects of inbreeding have also been explored extensively in *Drosophila menalogaster*. Collectively, it has been demonstrated that inbreeding in *D. melanogaster* reduces reproductive success by adversely affecting fertility, total egg count, larval survival, egg-to-adult viability and longevity of the flies [13,14,15,16,17,18,19]. Additionally, in most cases, the response of the flies to a variety of physical, chemical and biological stressors was found to be negatively affected by inbreeding [20,21,22,23]. However, very few studies have been conducted to monitor the effect of inbreeding on the behavior of *D. melanogaster*.

In *D. melanogaster*, full-sibling inbreeding of the outbred flies has been shown to reduce mating abilities of the males in a linear proportion, i.e., there is a strong negative correlation between the mating ability of males and the inbreeding coefficient [24]. Similarly, inbreeding in wild-type lines of *D. melanogaster* has also resulted in a noticeable drop in promiscuity of the inbred female flies compared to outbred flies [25]. Moreover, an increase in latency in mating was also observed in inbred flies. In total, this reduces the progeny count of inbred flies compared to the outbred flies [25]. Hoenigsberg and Santibanez also showed lower mating success in inbred males compared to outbred males and attributed it to low athletic ability and/or difficulty in interpreting the acceptance response in inbred flies [26]. Studies in relation to learning and locomotor activity showed more inter-line variations in inbred *D. melanogaster* [27,28]. However, Jorgensen et al. observed a cumulative decline in locomotor activity in the inbred flies compared to outbred flies [29].

Since behavior is an evolutionarily derived product underpinned by genetic and environmental factors, it plays a vital role in ensuring an organism’s survival and success [30,31,32]. Therefore, an in-depth investigation of the effects of inbreeding on innate behavioral traits is important. In this study, we have used *D.melanogaster* as a model organism to study the effects of inbreeding on photosensation and locomotor activity in both larvae and adult flies. Furthermore, the deviations in courtship, aggression, stress escape response and learning behaviors were also compared between outbred and inbred flies. Our findings demonstrate that inbreeding adversely affects many of the innate behaviors of *D. melanogaster*. However, better learning and an improved stress escape response were observed in the inbred fly population at the cost of the total population viability and reduced photosensation.

## 2. Materials and Methods

### 2.1. Base Population

The base population of wild-type *Drosophila melanogaster* used in the current study was established by approximately 100 pairs of *D. melanogaster* isolated in February 2019 from the wild in Karachi, Pakistan (24°55.73″ N, 67°7.99″ E). The base population was maintained on banana media at 25 ± 3 °C for a 10:14 light: dark photoperiod for over 150 generations by mass rearing.

### 2.2. Lineage Setup

Virgin fly pairs were randomly selected from the base population to set a total of 200 lineages in a single-pair conformation on agar media vials (2.5% agar with yeast paste placed on top). Each fly pair was considered to be a different lineage and was incubated at 25 ± 3 °C for a 10:14 light: dark photoperiod. The eggs were collected on day 2 and day 3, considering the pair placement day as day 0, to achieve a maximum egg count of 40 eggs per vial. The eggs were placed on standard cornmeal media (10.09% corn meal, 3.04% sucrose, 6.07% dextrose, 0.5% agar, 0.05% instant yeast and 0.125% methyl 4-hydroxybenzoate) and incubated at 25 ± 3 °C for a 10:14 light: dark photoperiod throughout the experiment. The cornmeal vials were then routinely observed for newly hatched flies for 20 days. From the onset of hatching, virgin flies were routinely collected every six hours. These virgin flies were then used to set up the experimental crosses. This strategy was uniformly applied during the entire course of investigation.

### 2.3. Experimental Crosses

**Figure 1:**
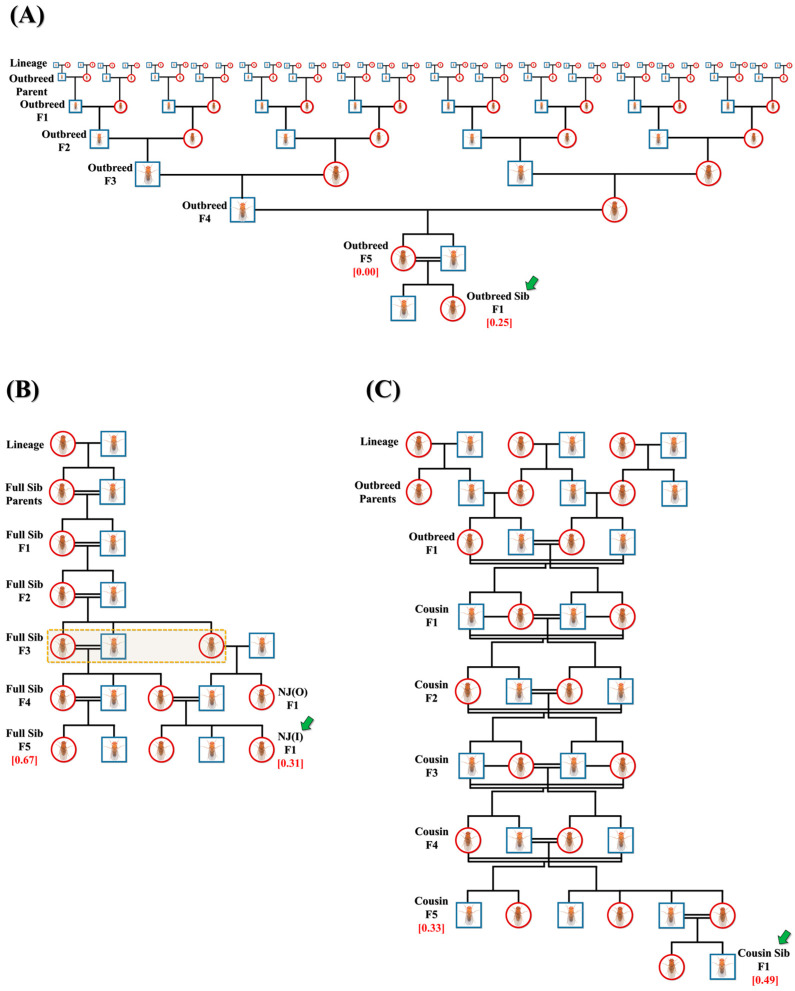
Representative pedigrees of crosses. Representative pedigrees of three main crosses: (**A**) outbred, (**B**) full-sibling and (**C**) cousin, to acquire larvae/flies with different inbreeding coefficients (red). Three additional crosses, namely, (**A**) outbred sibling (*f* = 0.25), (**B**) NJ(I) (*f* = 0.31) and (**C**) cousin sibling (*f* = 0.49), are also shown and indicated by green arrows. Here, females and males are depicted as red circles and blue squares, respectively. Dotted-line rectangle in (**B**) shows common grandparents of NJ(I).

Initially, multiple replicates of three major crosses were set in a single-pair confirmation, namely, outbred (*n* = 218) or full-sibling (*n* = 251) from lineages and cousin (*n* = 206) from outbred F1 progeny. The crosses were continued until F5 progeny to attain the final inbreeding coefficient (*f*) of 0.00 (outbred F5 progeny), 0.67 (full-sibling F5 progeny) and 0.33 (cousin F5 progeny) (Figure 1A–C). From the F1 to F5 generation, outbred crosses were set in a manner such that mating pairs never shared a common lineage (Figure 1A). From the F1 to F5 generation, all cousin crosses were planned to ensure that the mating pair would always be first cousins (Figure 1C). Multiple replicates of F5 progeny of outbred (*n* = 288) and cousin (*n* = 203) crosses were subjected to full-sibling crosses in a single-pair confirmation to attain F1 progeny with *f =* 0.25 (outbred sibling) and *f =* 0.49 (cousin sibling), indicated by green arrows in Figure 1A and C. In addition, F3 progeny of full-sibling crosses were set to mate with outbred F3 progeny of the opposite sex of different lineages in a pair confirmation. Resulting F1 progeny (NJ(O)) were mated with F4 progeny of full-sibling crosses in multiple replicates (*n* = 81), sharing ¾ common grandparents to attain new F1 progeny (NJ(I)) with *f =* 0.31, as highlighted by the green arrow in Figure 1B.

### 2.4. Egg-to-Adult Viability

The egg-to-adult viability of all crosses was deduced by dividing the total number of flies with the number of eggs placed per vial.

### 2.5. Behavioral Assays

Multiple behavioral assays were carried out on both larvae and adult flies (from different parents) with *f* = 0.00, 0.25, 0.31, 0.33, 0.49 and 0.67. All functional assays were conducted between ZT0 and ZT9. To perform larval assays, larvae were collected on day 5 of egg placement from different vials of representative crosses to maintain diversity. All larval assays were carried out in triplicate.

#### 2.5.1. Larval Crawling Assay

A larval crawling assay was performed as previously reported by Nichols et al. Briefly, five larvae were placed in the center of a 2% agarose plate, placed over a transparent sheet with 0.2 cm^2^ gridlines. Larval crawling was videotaped for a minute, and the total number of squares covered by the larvae in a minute were counted to assess their crawling ability [33]. A total of 24–75 larvae were assessed for each type of cross.

#### 2.5.2. Larval Phototaxis Assay

A larval phototaxis assay was conducted as reported by Lilly and Carlson [34]. The assay was performed in a four-quadrant plastic petri plate with two clear quadrants (1% agarose) and two dark quadrants (1% agarose containing 0.6% charcoal powder). In total, 30–60 larvae of different inbreeding coefficients were examined, and the response index (RI) for each set was calculated using the following formula:

$$RI=\frac{Number\;of\;larvae\;in\;dark\;quadrant-Number\;of\;larvae\;in\;light\;quadrant}{Total\;number\;of\;larvae\;assessed}$$

#### 2.5.3. Rapid Iterative Negative Geotaxis (RING) Assay

The assay was performed on 18–24 h mature virgin male and female flies as previously reported by Nichols et al. [33]. The assay was repeated five times, and all five photographs were analyzed using ImageJ software to measure the height climbed by individual flies. A minimum of 20 male and 20 female flies from each cross were examined.

#### 2.5.4. Phototaxis Assay

A phototaxis assay was performed on 24-hour-old virgin male flies as defined by Ali et al. with some modifications [35]. The flies were first starved for 6 h and then introduced individually into the dark chamber of a T-maze apparatus. After an acclimatization time of 30 s, the fly was allowed to move towards the light chamber. Flies that moved toward the light chamber within 10 s were considered positively phototactic and were subsequently used for the aversive phototaxis suppression assay. A minimum of 30 male flies of each cross were assessed for the phototaxis assay.

#### 2.5.5. Aversive Phototaxis Suppression Assay

The positively phototactic flies were used in this assay to assess their learning abilities as described by Ali et al. with some modifications [35]. Briefly, 1.3 mM quinine-sulfate (aversive stimulus)-soaked filter paper was placed into the light chamber of a T-maze apparatus. Ten conditioning trials were performed, each for one minute, to train the flies for movement against the light source in response to the aversive stimulus. After conditioning, test trials were performed, in which the flies were left for acclimatization (30 s) in a dark chamber and then allowed to move towards the light chamber. If the fly was observed in the light chamber within 10 s, it was declared as “Fail” and was scored as 0. If the fly retained the memory of the bitter-tasting quinine and avoided moving towards the light chamber for at least 10 s, it was considered to be a “Pass” and scored as 1 [35]. Testing trials were conducted five times, and a score was recorded each time. From each cross, a minimum of 18 male flies were assessed for the aversive phototaxis suppression assay.

#### 2.5.6. Forced-Swim Assay

A forced-swim assay was performed as described by Neckameyer and Nieto-Romero to observe the fly stress escape response [36]. The assay was carried out on 24–30-hour-old virgin flies. A single fly was placed in a well filled with 2 mL of 0.08% SDS solution. The fly was then videotaped for 5 min and analyzed for latency until first immobility and for the duration and number of immobility bouts. A minimum of 20 flies each of both genders were evaluated for all crosses.

#### 2.5.7. Aggression Assay

An aggression assay was conducted as described by Dierick on 5-day-old virgin males [37]. After eclosion, males were first placed in solitary confinement for 5 days. After this, a pair of males, progeny of the same type of cross, was placed in the fighting arena and covered with a clean cover slip. The flies were then videotaped for 20 min and evaluated for typical aggressive behaviors, including orientation, kicking, boxing, tussling, wrestling and wing charge. Latency to fighting, escalation in fighting frequency and fighting duration were monitored. For each cross, a minimum of 20 pairs of males were studied. The fighting index and fighting frequency were then calculated as follows:

$$Fighting\;Index=\frac{Total\;amount\;of\;time\;the\;males\;fly\;spends\;fighting}{Total\;duration\;of\;testing\;period\;(20\;\min)}$$

$$Fighting\;Frequency=\frac{Total\;number\;of\;pairs\;showed\;fighting}{Total\;numbers\;of\;pairs\;observed}\times 100$$

#### 2.5.8. Courtship Assay

A courtship assay was performed as described by Nichols et al. [33,38]. The assay was performed on 5-day-old virgin male and female flies that were placed in separate corn meal vials at 25 ± 3 °C for 5 days after eclosion. The assay was performed by placing a single male and female fly from a respective cross in a mating chamber, which was then covered with a clean cover slip. The flies were then videotaped for 20 min and evaluated for typical courtship behaviors such as orientation, tapping, licking, wing song and curling of the abdomen. Mating success, latency to courtship and copulation duration were monitored, and the courtship index was deduced using the following formula:

$$Courtship\;Index=\frac{Total\;time\;spent\;in\;courtship}{Total\;time\;spent\;in\;arena}$$

where courting time covers the duration from the exhibition of the first sign of courtship behavior until the start of mating. For each cross, a minimum of 20 pairs were assessed in this regard.

### 2.6. Statistical Analysis

Statistical analyses of the variables were conducted using GraphPad Prism v8.01. The nature of the data distribution was assessed using the Kolmogorov–Smirnov test (Supplementary File S1), and statistical significance between different groups was identified using the Kruskal–Wallis test and Dunn’s Test as a post-hoc test (Supplementary File S2). A linear regression analysis at a 95% confidence interval was also carried out to observe the association between two variables. In all cases, a *p* value less than 0.05 was considered statistically significant.

## 3. Results

### 3.1. Inbreeding Drops Egg-to-Adult Viability

**Figure 2:**
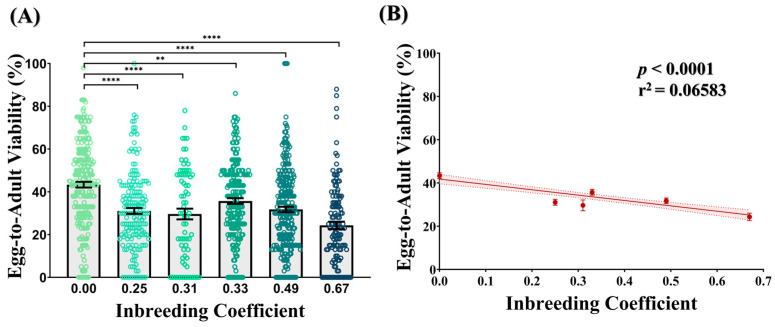
Effect of inbreeding on egg-to-adult viability. (**A**) Bar graph represents drop in egg-to-adult viability of inbred progeny (*f* > 0.00) flies compared to outbred flies (*f* = 0.00). The height of the bars represents the mean egg-to-adult viability, where error bars show standard error of mean. (**B**) Graph showing strong negative correlation between egg-to-adult viability and inbreeding coefficient. The solid and dotted lines represent the trend of the data and 95% confidence interval band, respectively. (** *p* ≤ 0.01, **** *p* < 0.0001; Kruskal–Wallis, Dunn’s Test.) For the total number of data points, kindly see Supplementary File S3.

Compared to the outbred crosses (*f* = 0.00), all crosses of different inbreeding coefficients showed a 1.21- to 1.78-fold drop in the egg-to-adult viability. In all cases, this drop was found to be statistically significant (Figure 2A). Consistently, a decline in the egg-to-adult viability was observed with an increasing inbreeding coefficient (Figure 2B), representing a strong inbreeding depression.

### 3.2. Inbreeding Adversely Affects Larval Crawling Ability

**Figure 3:**
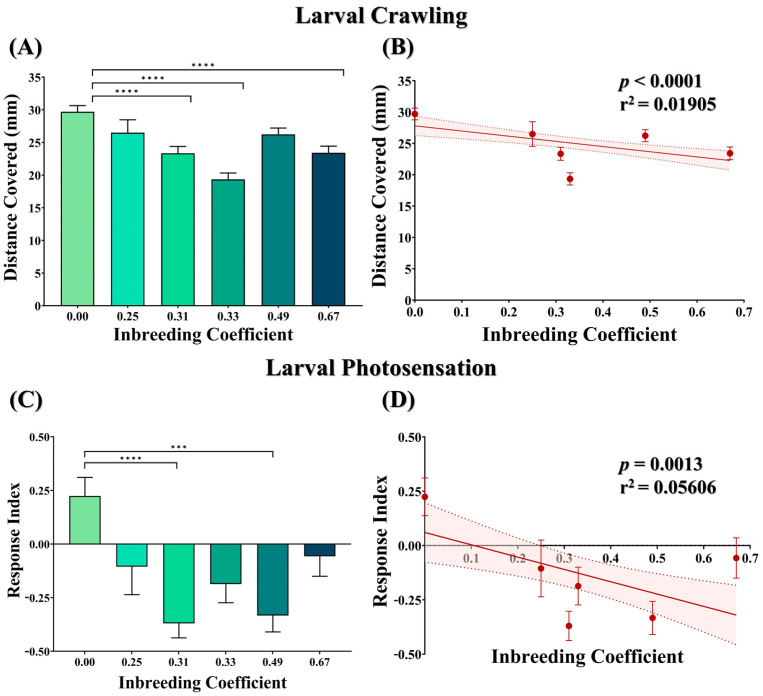
Effect of inbreeding on larval locomotory and phototaxis. (**A**) Bar graph showing comparison between the distance covered by the larvae of different inbreeding coefficients. (**B**) Graph showing correlation between distance covered and inbreeding coefficient. Graphs showing (**C**) comparison of phototaxis response index and (**D**) correlation between inbreeding coefficient and phototaxis response index. Here, the height of the bars represents the mean values, and error bars show standard error of mean. The solid and dotted lines represent the trend of the data and 95% confidence interval band, respectively. (*** *p* ≤ 0.001, **** *p* ≤ 0.0001; Kruskal–Wallis, Dunn’s Test.) For the total number of data points, kindly see Supplementary File S3.

F5 larvae of outbred crosses were found to be more motile compared to the larvae of all inbred crosses. This decrease in the larval motility was found to be statistically significant in all cases except for the larvae of the outbred sibling cross (*f* = 0.25) and the cousin sibling cross (*f* = 0.49) (Figure 3A). Similarly, with increased inbreeding, a coefficient decrease in the larval motility was observed (Figure 3B).

### 3.3. Inbreeding Alters Larval Innate Phototaxis Response

The phototactic behavior of the larvae was measured in terms of the response index (RI), where a positive RI value represents innate negative phototaxis, and a negative RI value represents aberrant positive phototaxis in larvae. Compared to the outbred cross, a statistically significant decrease in larval innate phototaxis response was found in NJ(I) F1 (*f* = 0.31) and cousin siblings (*f* = 0.49) (Figure 3C). Nevertheless, the phototaxis response in larvae was found to be adversely affected with increased inbreeding (Figure 3D).

### 3.4. Inbreeding Adversely Affects Fly Climbing Ability

**Figure 4:**
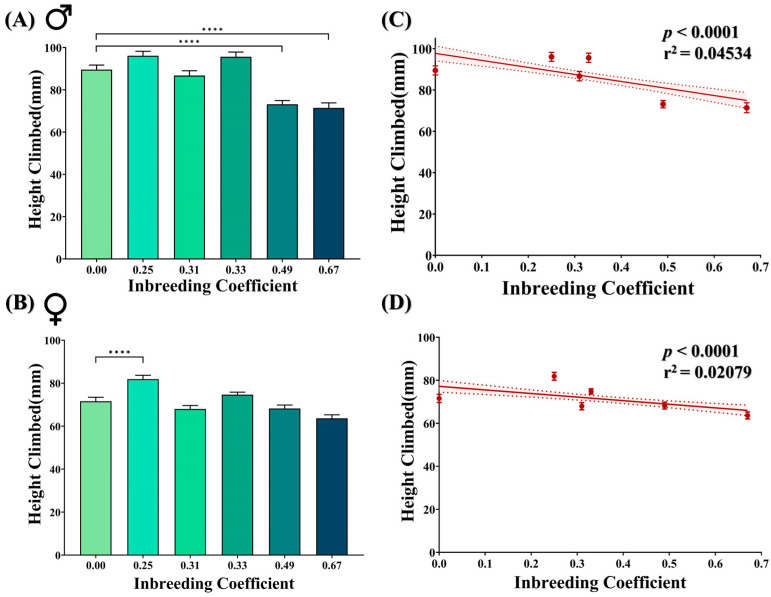
Effect of inbreeding on negative geotaxis. Bar graphs showing difference in the height climbed by (**A**) male and (**B**) female flies of different inbreeding coefficients. Graphs showing correlation between inbreeding coefficient and height climbed by both (**C**) male and (**D**) female flies. The height of the bars represents the mean height climbed by flies, where error bars show standard error of mean. Solid and dotted lines represent trend of the data and 95% confidence interval band (**** *p* ≤ 0.0001; Kruskal–Wallis, Dunn’s Test). For the total number of data points, kindly see Supplementary File S3.

Negative geotaxis is a natural behavior of adult flies. Marginal improvement has been observed in inbred female flies of *f* = 0.25 compared to F5 progeny of the outbred cross. However, at higher inbreeding levels, a noticeable drop in the climbing activity was observed in inbred male flies of *f* = 0.49 and *f* = 0.67 (Figure 4A,B). Nevertheless, in total, with an increasing inbreeding coefficient, the climbing activity of both male and female flies appeared to be negatively affected (Figure 4C,D).

### 3.5. Inbreeding Negatively Influences Positive Phototactic Behavior of Flies

**Figure 5:**
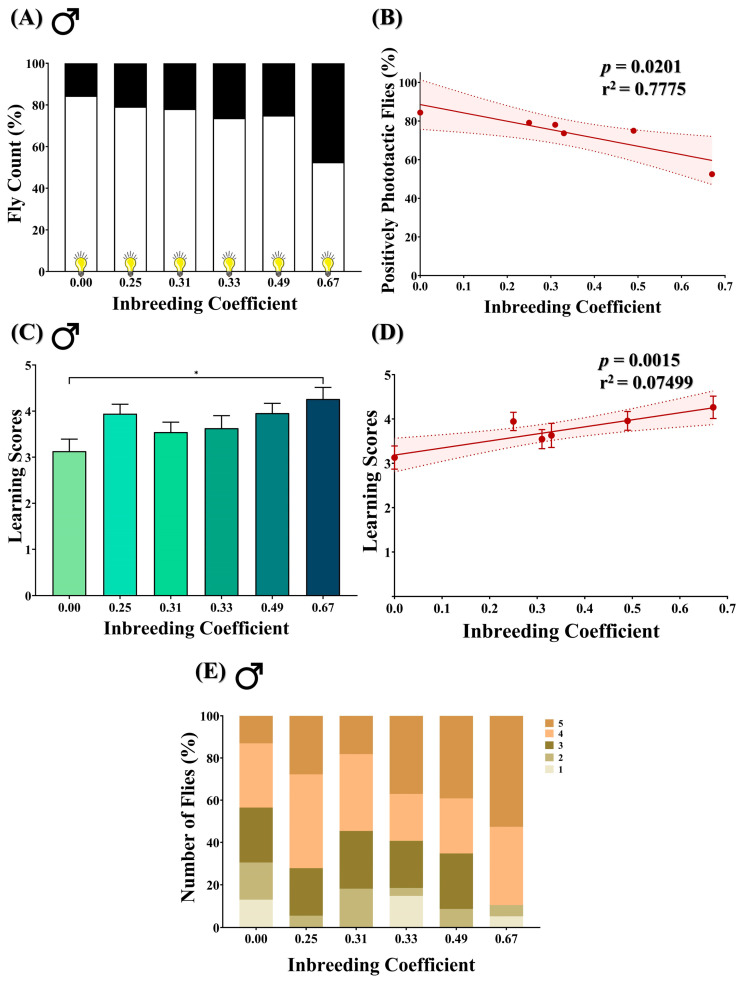
Effect of inbreeding on phototactic behavior and learning ability in flies. (**A**) Stacked bar graph representing the percentage of positive and negative phototactic flies with different inbreeding coefficients. (**B**) Graph showing correlation between the percentage of positively phototactic flies and inbreeding coefficient. (**C**) Bar graph representing comparison between the learning scores of positively phototactic flies of different inbreeding coefficients. (**D**) Linear regression graph showing the positive correlation between learning scores and inbreeding coefficient. (**E**) Stacked bar graph showing the distribution of male flies with different inbreeding coefficients according to the learning scores, where 1 is the lowest and 5 is the highest. The height of the bars represents the mean values, where error bars show standard error of mean. Solid and dotted lines represent trend of the data and 95% confidence interval band, respectively (* *p* < 0.05; Kruskal–Wallis, Dunn’s Test). For the total number of data points, kindly see Supplementary File S3.

A T-maze assay was performed on male flies to access their innate positive phototactic response. In outbred F5 progeny (*f* = 0.00), 84.4% of flies were observed to be positively phototactic. In comparison, in inbred progeny, the number of positively phototactic flies dropped to 53.5% (*f* = 0.67) (Figure 5A). A decline in the number of positively phototactic flies was consistently observed with an increase in the inbreeding coefficient (Figure 5B).

### 3.6. Inbreeding Improves Learning in Positively Phototactic Flies

The aversive phototaxis suppression assay was performed to evaluate the learning ability of positively phototactic male flies. The assay generated five scores for each fly, ranging from 1 to 5, where each score indicates the number of times the fly passed the learning test. In comparison to outbred progeny, an increase in the mean learning score was found in inbred flies of *f* = 0.67 (Figure 5C), with an overall improvement in the learning score with an increased inbreeding coefficient (Figure 5D). Interestingly, the highest score of “5” was achieved by 13% of flies in outbred F5 progeny (*f* = 0.00), which increased to 53% in inbred flies (*f* = 0.67) (Figure 5E).

### 3.7. Inbreeding Influences Stress Escape Response

**Figure 6:**
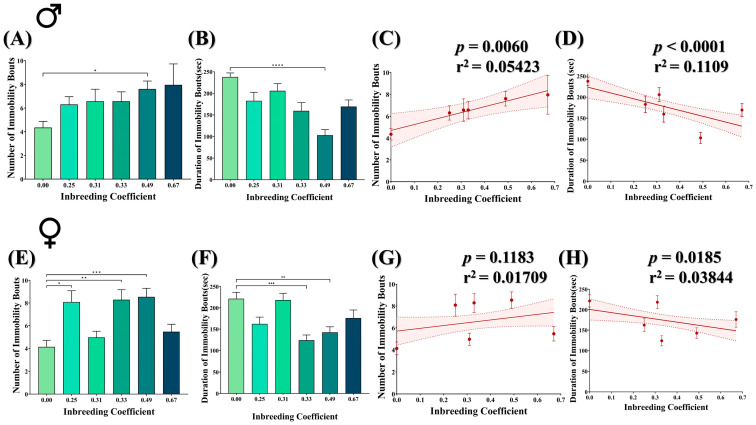
Effect of inbreeding on stress escape response of flies. Bar graphs representing comparison in (**A**) number and (**B**) duration of immobility bouts in male flies with different inbreeding coefficient. Graphs showing correlation between inbreeding coefficient and (**C**) number and (**D**) duration of immobility bouts in male flies, respectively. Similarly, bar graphs representing comparison in (**E**) number and (**F**) duration of immobility bouts in female flies with different inbreeding coefficients. Graphs showing correlation between inbreeding coefficient and (**G**) number and (**H**) duration of immobility bouts in female flies, respectively. The height of the bar represents the mean value for observed data, where error bars show standard error of mean. The solid and dotted lines represent the trend of the data and 95% confidence interval bands, respectively. (* *p* < 0.05, ** *p* ≤ 0.01, *** *p* ≤ 0.001, **** *p* ≤ 0.0001; Kruskal–Wallis, Dunn’s Test.) For the total number of data points, kindly see Supplementary File S3.

The forced-swim assay was conducted to evaluate the escape response of both male and female flies under stress conditions. The flies were examined for the number and duration of immobility bouts, where physical immobility represents behavioral despair in flies. In general, both male and female inbred flies showed a better escape response compared to outbred flies. For example, the number of immobility bouts was increased by nearly twofold in male inbred flies (*f* = 0.49) compared to outbred flies (Figure 6A), with a significant decrease in the duration of immobility bouts (Figure 6B). Consistently, in male flies, an increased inbreeding coefficient showed an increase in the number of immobility bouts (Figure 6C) but a decrease in the duration of immobility bouts (Figure 6D). This collectively represents a longer duration of mobility in the inbred flies than the in outbred flies under stress conditions. Although female inbred flies also showed an increase in the number of immobility bouts (Figure 6E) and a decrease in the duration of immobility (Figure 6F), the overall effect is less pronounced compared to male inbred flies (Figure 6G,H).

### 3.8. Inbreeding and Aggression in Flies

**Figure 7:**
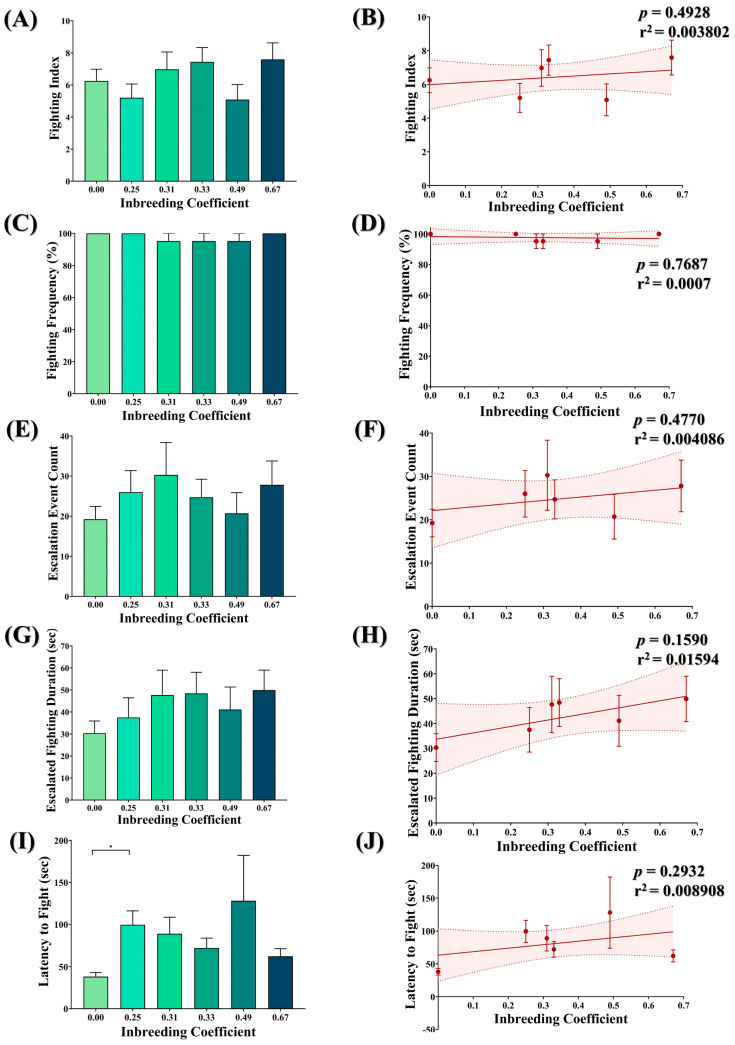
Effect of inbreeding on aggression. Graphs showing comparison and correlation of (**A**,**B**) fighting index, (**C**,**D**) fighting frequency, (**E**,**F**) count of escalated fighting event, (**G**,**H**) duration of escalated fighting and (**I**,**J**) latency to fight, respectively, with reference to inbreeding coefficient. The height of the bar represents the mean value for observed data, where error bars show standard error of mean. The solid and dotted lines represent the trend of the data and 95% confidence interval bands, respectively. (* *p* < 0.05; Kruskal–Wallis, Dunn’s Test.) For the total number of data points, kindly see Supplementary File S3.

No significant difference was observed between the fighting index (Figure 7A,B), fighting frequency (Figure 7C,D), escalated fighting events (Figure 7E,F) and escalated fighting duration (Figure 7G,H) of inbred flies compared to outbred flies. The only exception is the marginal increase observed in the latency to fight in inbred flies compared to outbred flies (Figure 7I). However, again, this has no association with the inbreeding coefficient (Figure 7J).

### 3.9. Inbreeding Adversely Affects Courtship Behavior in Flies

**Figure 8:**
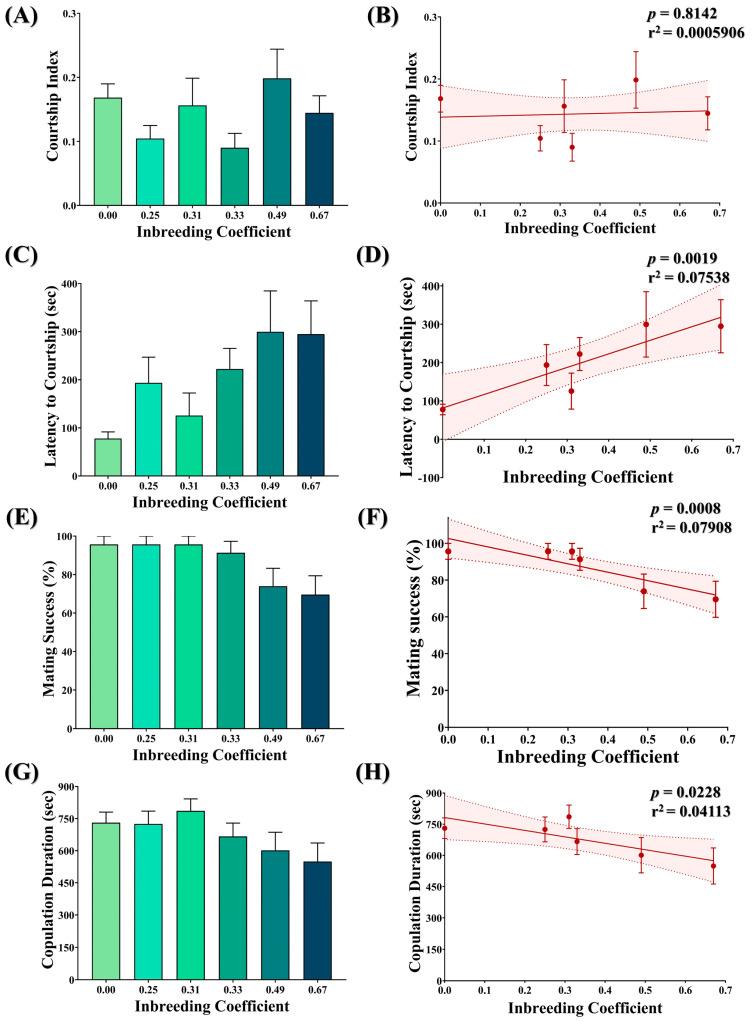
Effect of inbreeding on courtship behavior. Graphs showing comparison and correlation of (**A**,**B**) courtship index, (**C**,**D**) latency to courtship, (**E**,**F**) mating success and (**G**,**H**) copulation duration, respectively, with reference to inbreeding coefficient. The height of the bar represents the mean value for observed data, where error bars show standard error of mean. The solid and dotted lines represent the trend of the data and 95% confidence interval bands, respectively (Kruskal–Wallis, Dunn’s Test). For the total number of data points, kindly see Supplementary File S3.

No statistically significant difference was observed in the courtship index of flies with different inbreeding coefficients (Figure 8A). Consistently, no association was observed between the inbreeding coefficient and the courtship index (Figure 8B). Nevertheless, an increase in the latency to courtship (Figure 8C,D), a drop in mating success (Figure 8E,F) and a drop in copulation duration (Figure 8G,H) were observed with an increased inbreeding coefficient.

## 4. Discussion

The overall biological fitness of an organism is a collective manifestation of several factors, and an organism’s behavior is essentially one of the important biological factors in this regard. *Drosophila* and higher animals, including humans, share considerable behavioral similarities. Therefore, the fruit fly is often used to study behavioral changes in response to varying biological, physical and chemical conditions [28,39,40]. Similarly, the effect of inbreeding has been extensively explored in *Drosophila melanogaster* with reference to reproductive fitness and stress response [13,14,15,16,17,18,19,20,21,22,23,24,25,26]. Considering the genetic basis of behavior, inbreeding could influence the organism’s behavior through the accumulation of certain traits over generations. However, very few studies have been conducted on *D. melanogaster* in this regard, and existing studies are mostly focused on courtship and locomotor activities of adult flies [24,25,26,28,29]. In this study, we demonstrate how different levels of inbreeding influence seven of the key innate behaviors of *D. melanogaster*.

To the best of our knowledge, this is the first study that explores the effect of inbreeding on the behavior of *D. melanogaster* larvae. Our study demonstrates that in *D. melanogaster* larvae, photosensation and locomotion are negatively influenced by an increased intensity of inbreeding (represented by the inbreeding coefficient) (Figure 3). The larval stage of *D. melanogaster* is nutritionally demanding and requires continuous foraging, where sensations of light and odor as well as locomotion hold profound importance [41,42,43,44]. Therefore, aberration in these traits could lead to a decline in the transformation of larvae into adult form. Consistent with this possibility, we also observed a substantial drop in the egg-to-adult viability with an increased inbreeding coefficient (Figure 2). Altogether, this suggests that inbreeding depression could be monitored even at the larval stages, where key behaviors required for survival and/or the subsequent transformation of larvae are adversely affected by inbreeding.

We observed that the adverse impact of inbreeding on photosensation and locomotion of *D. melanogaster* larvae continued to the adult stage, as represented by the statistically significant decline in climbing activities and the number of positively phototactic flies in the inbred population (Figure 4 and Figure 5A). Negative geotaxis and positive phototaxis are two of the fundamental behaviors that are often used as an indicator of general fitness in flies [45,46]. Disturbances in locomotion and/or photosensation have been shown to affect the circadian clock, the acquisition of food and of mates and the ability to escape from predators in a number of different animals, including humans and *D. melanogaster* [47,48,49,50]. Moreover, abnormal photosensation has also been used as a marker for disturbance in neuroanatomy and/or neurophysiology in *D. melanogaster* [51,52]. Thus, our data show that inbreeding adversely affects two of the fundamental traits of *D. melanogaster*, which may consequently challenge the survival and extension of the gene pool of an inbred population. Although a drop in photosensation implies potential defects in the neuroanatomy and/or neurophysiology of inbred flies, surprisingly, in our study, the positively phototactic male inbred flies with higher genomic homozygosity showed better learning ability compared to the outbred flies (Figure 5C,D). This apparent conundrum could be due to the fact that only positively phototactic flies are studied for learning behavior, and this interesting observation is the collective manifestation of both natural and artificial selection. For example, continuous inbreeding has led to a reduction in genetic heterogeneity in the base population, where the fly population was first negatively selected for positive phototactic activity (Figure 5A,B). However, a small subset of this population with better phototactic activity also exhibit improved learning on assessment (Figure 5C,D). This demonstrates that inbreeding could improve certain traits such as learning in a subsection of the population but at the cost of dwindling reproductive fitness (reduced egg-to-adult viability) of the total population. Previously, Nepoux et al. explored the effects of 12 generations of sibling mating on aversive learning through olfactory avoidance and showed no detectable decline in learning ability in moderately inbred lines (*f* = 0.38) [27]. Consistent with our findings, they also reported highly variable learning performance in strongly inbred lines.

A dimorphic stress response has been observed against different biological and chemical stressors in *D. melanogaster* [22,53,54]. Our findings of the forced-swim assay collectively suggest that inbreeding may have improved the stress escape response in male but not in female inbred flies (Figure 6). Many studies have concluded that inbreeding in *D. melanogaster* reduces its tendency to resist against physical [22], biological [23] and chemical stresses [55]. However, some studies have also demonstrated that early-life exposure to different stressors (heat, ethanol, competition and bacterial exposure) reduces inbreeding depression on post-stress reproductive fitness in adult flies compared to those that were grown in a benign environment [56,57]. Moreover, it is also possible that different levels of inbreeding may result in different outcomes, where purging could occur in certain cases for the deleterious traits related to the stress response in inbred flies. However, these findings cannot be generalized, as dimorphism in the stress response also varies for different stressors [22,53,54].

Like many other behaviors, aggression has a genetic basis [58]. For example, mutants (black) of *D. melanogaster* have reduced levels of β-alanine and were found to be less aggressive than wild-type flies [59]. Similarly, aggression analysis in isogenic mutant lines for *tramtrack*, *Darkener of apricot*, *longitudinal lacking* and *scribbler* showed varying levels of aggression [59]. Moreover, a higher level of inbreeding was also reported to decrease aggression in mice [60]. Although we have not observed any peculiar change in the aggressive behavior between flies of different inbreeding coefficients, a significant delay in the initiation of fighting was observed in inbred flies compared to outbred flies (Figure 7I). Aggressive behavior plays a significant role in securing resources such as food and territory, competing for mates, defending against predators and shaping social interaction [32]. However, aggression entails an energy-consuming process and may harbor a potential threat to survival. Nevertheless, innate aggressive behavior in flies is essentially passed through the processes of natural selection and is considered to be evolutionarily advantageous [32]. Therefore, a delay in the exhibition of aggression, as observed in the inbred flies, may compromise their fitness.

We observed an increase in the latency to courtship and a decline in mating success and copulation duration in inbred flies compared to outbred flies (Figure 8). Our observation is consistent with the findings of earlier studies in which the adverse effects of inbreeding on male mating ability have been demonstrated [24,61,62,63,64,65]. For example, Miller et al. assessed six isogenic lines for the second chromosome and observed an increase in the latency to courtship and a decrease in male mating ability [66]. Similarly, a linear decline has been observed in male competitive mating ability for 18 generations of full-sibling crosses [24]. Moreover, Averhoff et al. demonstrated that infertility within inbred lines is mostly due to failure in mating. They further showed that inbred flies are deficient in sensing the sexual stimuli (pheromones) that play a key role in initiating courtship [62]. Collectively, this suggests that inbreeding negatively influences the mating ability of *D. melanogaster*.

Behavior is one of the strongest determining factors in the evolutionary success of the organism [67,68]. During the last three decades, it has been well-demonstrated that allelic variations lead to phenotypic differences in behavior [69]. Consistently, many behaviors of *D. melanogaster* are firmly tagged with a gene and/or its alleles. For example, *CG9498*, *dpr6*, *Lrrk* and *numb* are linked with climbing ability in flies [70]. Moreover, the *trp* gene mutant has shown reduced negative geotaxis in flies [71]. Likewise, mutants for *w*, *norpA*, *ninaE*, *ora*, *rdgA*, *rdgC*, *tro*, *so*, *Ppb*, *sev* and *gl* have shown abnormal photosensation in adult flies [72,73]. Several genes are also linked with different courtship behaviors such as courtship initiation (*fru* and *Tre1*) [74], male courtship intensity (*crl*, *cuc*, *hni* and *pale*) [75,76,77,78], female receptivity (*spin* and *dsf*) [79,80] and courtship song (*cac* and *per*) [81,82]. Mutants of genes such as *dunce*, *rutabaga*, *dumb*, *lio* and *lat* have shown poor learning and memory retention in both olfactory- and photosensation-based learning assays [83,84]. Since inbreeding results in the transgenerational accumulation of both deleterious and/or advantageous alleles, the findings of the present investigation could be explained in similar terms. On the one hand, the accumulation of certain alleles leads to inbreeding depression in most innate behaviors. However, inbreeding may also improve some of behaviors such as learning and stress escape response, but with noticeable gene purging, as reflected by the inbreeding depression in egg-to-adult viability (Figure 2). It is important to mention that the laboratory line used in this study has been maintained for over 150 generations. Therefore, it is likely that it has undergone inbreeding resulting in the fixation of certain behavioral traits. However, to maximize genetic heterogeneity, instead of jar-to-jar transfer, progeny of multiple jars was collected and mixed together to create a new jar of a founder population during the whole course of line maintenance (>150 generations). Moreover, most studies in which the effect of inbreeding has been investigated in *D. melanogaster* indigenously developed and used lines of fruit flies that were maintained under laboratory conditions for many years [27,28,29]. Nevertheless, it is interesting to explore the nature of alleles in inbred flies that have accumulated over generations due to inbreeding. Additionally, the identification of orthologues of these genes in humans and other mammals may extend the findings in relation to humans and livestock [85,86].

## 5. Conclusions

Inbreeding in humans and animals has been firmly associated with a variety of anatomical, behavioral and physiological aberrations [6,7,8,9]. In this regard, *Drosophila melanogaster* has been effectively used to study the influence of inbreeding on reproductive fitness and stress tolerance, but very few studies have explored the effects of inbreeding on the innate behavior of flies. Herein, we have demonstrated that inbreeding adversely influences five of the seven assessed innate behaviors of *D. melanogaster* both in larvae and adult flies. Given the genomic and behavioral similarities between *Drosophila* and larger animals including humans, the findings could further our understanding of the genetics that underpin behavior in humans and other livestock, where inbreeding exists in different forms and shapes.

## Data Availability

Not applicable.

## Supplementary Materials

The following supporting information can be downloaded at: https://www.mdpi.com/article/10.3390/biology12070926/s1, File S1: Results of Kolmogorov–Smirnov test; File S2: Kruskal–Wallis, Dunn’s Test; File S3: Sample Size of Larvae and Flies.
